# Editorial: Proceedings of the 3rd ISESSAH Conference 2019

**DOI:** 10.3389/fvets.2021.807796

**Published:** 2021-11-25

**Authors:** Dustin L. Pendell, Thomas L. Marsh, Bouda Vosough Ahmadi, Didier Raboisson, Henk Hogeveen, Jonathan Rushton

**Affiliations:** ^1^Department of Agricultural Economics, Kanas State University, Manhattan, KS, United States; ^2^School of Economic Sciences and Paul G. Allen School for Global Health, Washington State University, Pullman, WA, United States; ^3^The European Commission for the Control of Foot and Mouth Disease, Animal Production and Health Division, Food and Agricultural Organization, Rome, Italy; ^4^Université de Toulouse, ENVT, Toulouse, France; ^5^Business Economics Group, Wageningen University and Research, Wageningen, Netherlands; ^6^Institute of Infection, Veterinary and Ecological Sciences, University of Liverpool, Liverpool, United Kingdom

**Keywords:** economics, social sciences, ISESSAH, animal diseases, zoonosis

## Introduction

Global animal health and welfare challenges are multifaceted and growing in complexity with the growth of human and animal populations accompanied with significant climate and environment changes. There is a realization that linkages between animal, human, and environmental health are strong and need animal and human health professionals to work together to address these growing, complex issues. This proceeding presents work that explores this discussing transboundary animal diseases [like African swine fever (ASF), foot-and-mouth disease (FMD), highly pathogenic avian influenza (HPAI)], zoonotic diseases (like rabies, liver flukes), antibiotic residues, climate change, and many more animal health and welfare issues. These papers and the ideas and work in them were presented orally and as posters at the third annual conference of the International Society for Economics and Social Sciences of Animal Health (ISESSAH) held in Atlanta, Georgia, United States in August 2019. The conference was held in conjunction with the Agricultural and Applied Economics Association's annual meeting.

The aim of the conference was to highlight interactions between human behavior and animal health, decision making impacts on biosecurity, and the One Health approach for evaluation. The proceedings of the third ISESSAH conference focus on how economics and social sciences modeling in animal health and food production can support animal and zoonotic disease prevention, mitigation, and eradication. There are 19 papers in total, including 16 original research articles, two systematic reviews, and one perspective. The three themes in this Research Topic are: (1) decision support economic modeling in animal health and food production; (2) economic assessment of infectious animal diseases and zoonoses and related control; and (3) evaluation of animal health and welfare issues.

## Decision-Support Economic Modeling in Animal Health and Food Production

Kappes and Marsh estimated the protein, lipid, and carbohydrate macronutrient consumption from household food consumption in western Kenya. The authors demonstrate that livestock illness is associated with increased macronutrient shadow prices, and hence the costs of available energy consumption. Dennis et al. used two behavioral frameworks, Random Utility and Regret Minimizing, to compare demand elasticities and willingness to pay in response to an *E. coli* or antibiotic residue recall. They found the regret minimizing framework to be more powerful when assessing consumer responses. Clark et al. investigated the risks associated with producers balancing the costs of biosecurity investments and the expected benefits of those investments. Using an online experimental game that simulates biosecurity investment allocation of a pork production facility during an outbreak, they did not find any significant differences between the risk behaviors and biosecurity investments decisions of the industry professionals and the non-industry participants. Iles et al. incorporated human memory and rationality into an agent-based modeling framework to evaluate producers' decision making to vaccinate cattle in Kenya. The authors concluded that memory and rationality parameters successfully differentiated between vaccination decisions that are annual and once-for-life. de Menezes et al. adopted a Social Network Analysis and conducted an exploratory analysis of cattle movement in Brazil. They found cattle movement networks were strongly connected, suggesting a high-speed diffusion of FMD, if reintroduced. Additionally, they concluded the need for investment in animal movement, education for producers and technologies to assist in early detection, diagnosis and eradication of FMD outbreaks. Pramuwidyatama et al. used Theory of Planned Behavior to better understand the factors associated with small-scale broiler producers in Western-Java toward cleaning and disinfection, vaccination, reporting, and stamping out in the event of HPAI. The authors suggested policies should be emphasized toward preventative measures rather than control measures. Aslam et al. employed key informant interviews and a focus group discussion to characterize and map the broiler and layer production systems, values chains, and poultry disease management in Pakistan.

## Economic Assessment of Infectious Animal Diseases and Zoonoses and Related Control Measures

Thomann et al. assessed the profitability of porcine reproductive and respiratory syndrome vaccines in Germany. The authors found the benefits were greatest when both sows and piglets were vaccinated and when vaccination was adopted by previously non-vaccinating herds. Ozturk et al. utilized a partial budget approach to analyze the economic impacts of biannual mass vaccination vs. vaccination every 4 months for FMD in border cities in Turkey. They conclude that the more intense vaccination strategy could be more cost effective than the current biannual mass vaccination. Machado Junior et al. used a Bayesian hierarchical spatio-temporal model to determine the factors associated with farm and broiler house characteristics and management practices using data from a Brazilian integrated broiler enterprise. The authors suggested that both time and space increase the odds of isolating *Samonella* spp. from litter, as well as, the size and type of the broiler house, total housing area per farm, and the number of litter recycles. Gilbert et al. evaluated the economic impacts of coccidiosis under different efficacies on control in European intensive broiler systems. The authors concluded that the impacts of coccidiosis increased rapidly as control efficacy decreased. Niemi analyzed how ASF outbreaks impacted swine production (quantity and prices) and exports in 11 European countries using a seemingly unrelated regression. He found that new ASF cases reduced production of pork by 4% and exports by 15% in the following year after the outbreak, and 3–4% in the national pig inventory. In a perspective by Beyene et al., they provided evidence on the socioeconomic burden of rabies in dogs in Ethiopia. Shrestha et al. investigated the financial impacts of liver fluke infections with and without climate change effects on Scottish livestock farms using a linear programming model. The authors found a 12 and 6% decrease in net profit on an average dairy and beef, respectively, farm under normal disease conditions and 2- and 6-fold losses in dairy and beef, respectively, farms when climate changes effects are incorporated into the model.

## Evaluation of Animal Health and Welfare Issues

Thompson et al. explored the effects violence and environmental effects along the U.S.—Mexico border on cattle fever ticks. The authors suggest the both media-reported violence and changes in weather impact the rate at which infested cattle are apprehended. Rothman-Ostrow et al. evaluated the use of Tropical Livestock Unit to measure biomass and compare that to two proposed alternatives. After analyzing the three methods using publicly available data for cattle from six sub-Saharan Africa countries, the authors highlight the difference in results between the three methods and suggest that standardizing data collection will allow for better livestock population and biomass estimates. By conducting a systematic literature review and meta-analysis, Afonso et al. estimated the frequency levels of lameness in British dairy cattle and documented the patterns of how lameness is detected and classified in research. They concluded that regardless the method that was used to measure lameness, it is high in British dairy cattle. In an article by Raboisson et al., they used a meta-analysis to look at losses due to clinical mastitis losses and identify which factors influence those effects. The average loss was estimated at €224 per case and that labor, drug, and culling costs, and treatment price had a significant impact on the losses. Using collected blood samples and surveys from herd management administrators on pig farms in Indonesia, Nurhayati et al. estimated and investigated which risk factors impacted Swine influenza virus (SIV) seropositivity status. The authors found farm-level SIV seropositive rate was 26% and the presence of animals on the farm (excluding pigs), keeping breeding sows for <2 years, being located near a poultry farm, and purchasing pigs only through collectors increased the risk of being seropositive to SIV.

## Conclusion

The collection of 19 articles in this Research Topic from the 3rd annual ISESSAH conference provides a good read on important socioeconomic issues surrounding animal health management and welfare. It is the hope from the authors of this Editorial that ISESSAH and similar organizations will continue to bring animal health professionals together to tackle the growing complex animal health issues that we are faced in today's world, ultimately increasing societal welfare.

## Author Contributions

DP drafted the editorial. TM and DP had an advisory role and provided input at the designing stage of the Research Topic. TM, JR, DR, HH, and BV reviewed and revised the editorial. DP, BV, DR, HH, and JR contributed to the reviewing and editing of the papers published in the proceedings of the 3rd ISESSAH conference 2019. All authors contributed to the article and approved the submitted version.

## Funding

This work was partially supported by the U.S. Department of Agriculture (USDA) NIFA Award No. 2017-67023-26266.

## Conflict of Interest

The authors declare that the research was conducted in the absence of any commercial or financial relationships that could be construed as a potential conflict of interest.

## Publisher's Note

All claims expressed in this article are solely those of the authors and do not necessarily represent those of their affiliated organizations, or those of the publisher, the editors and the reviewers. Any product that may be evaluated in this article, or claim that may be made by its manufacturer, is not guaranteed or endorsed by the publisher.

